# MR‐DELTAnet: A Longitudinal MRI‐Transformer Model Predicting Pathological Complete Response and Revealing Immune Microenvironment via scRNA‐seq in Locally Advanced Rectal Cancer

**DOI:** 10.1002/advs.202517721

**Published:** 2025-12-19

**Authors:** Wuteng Cao, Huaxian Chen, Jiao Li, Yihui Zheng, Liqing Xie, Guozhong Xiao, Zeyan Wang, Fen Yuan, Junhong Chen, Chongbao Sun, Jing Dai, Jinping Zeng, Xinhua Wang, Lei Wu, Hongcheng Lin

**Affiliations:** ^1^ Department of Radiology, The Sixth Affiliated Hospital Sun Yat‐sen University No.26, Yuancun Er Heng Road, Tianhe District Guangzhou Guangdong 510655 China; ^2^ Guangdong Provincial Key Laboratory of Colorectal and Pelvic Floor Diseases, Guangdong Research Institute of Gastroenterology, The Sixth Affiliated Hospital Sun Yat‐sen University No.26, Yuancun Er Heng Road, Tianhe District Guangzhou Guangdong 510655 China; ^3^ Biomedical Innovation Center, The Sixth Affiliated Hospital Sun Yat‐sen University No.26, Yuancun Er Heng Road, Tianhe District Guangzhou Guangdong 510655 China; ^4^ Department of Coloproctology, The Sixth Affiliated Hospital Sun Yat‐sen University No.26, Yuancun Er Heng Road, Tianhe District Guangzhou Guangdong 510655 China; ^5^ Department of Radiology Longyan First Affiliated Hospital of Fujian Medical University No.105, Jiuyi North Road, Xinluo District Longyan City Fujian 364028 China; ^6^ Department of Radiology Zhongshan City People's Hospital No.2 Sunwen East Road Zhongshan City Guangdong 528403 China; ^7^ Department of Radiology Jiangsu Provincial Hospital of Chinese Medicine No.155 Hanzhong Road, Qinhuai District Nanjing JiangSu 210029 China; ^8^ BGI Research No. 9 Yunhua Road, Meisha Street, Yantian District Shenzhen Guangdong 518085 China; ^9^ Department of Radiology, Guangdong Provincial People's Hospital (Guangdong Academy of Medical Sciences) Southern Medical University No.106, Zhongshan 2nd Road Guangzhou Guangdong 510080 China; ^10^ Guangdong Provincial Key Laboratory of Artificial Intelligence in Medical Image Analysis and Application No.106, Zhongshan 2nd Road Guangzhou Guangdong 510080 China

**Keywords:** deep learning, locally advanced rectal cancer, neoadjuvant chemoradiotherapy, pathological complete response

## Abstract

Accurate tumor response assessment to neoadjuvant chemoradiotherapy (NCRT) is crucial for personalized treatment strategies in locally advanced rectal cancer (LARC). However, reliable non‐invasive assessment tool remains clinically lacking. To fill this unmet need, MR‐DELTAnet, a longitudinal MRI‐based Transformer framework that integrates Delta‐Efficient Latent‐Temporal Attention, is constructed to predict pathological complete response (pCR) to NCRT in locally advanced rectal cancer patients. In a multicenter retrospective cohort of 1,026 LARC patients between July 2012 and July 2023, MR‐DELTAnet demonstrated robust discriminative performance across independent datasets, with the area under the curves (AUC) of 0.93 (95% CI 0.90‐0.96), 0.88 (95% CI 0.82‐0.94) and 0.90 (95% CI 0.79‐1.00) and in training (n═633), internal validation (n═212) and external validation (n═181) sets, respectively. Risk‐stratification by MR‐DELTAnet prediction scores reveals significant survival differences: low‐score patients exhibit prolonged disease‐free and overall survival versus high‐score patients (log‐rank *p*<0.05). Applying the model to an independent single‐cell RNA sequencing cohort (n═26) discloses biologically distinct immune microenvironments: high‐score tumors are myeloid‐rich and immunosuppressive, whereas low‐score tumors harbor cytotoxic T‐cell‐dominant. Clinically, MR‐DELTAnet provides an accurate, non‐invasive tool for preoperative identification of pCR likelihood and biological phenotype, thereby potentially informing individualized treatment strategies for LARC management.

## Graphical abstract

The MR‐DELTAnet model utilizes longitudinal MRI to predict treatment response after neoadjuvant chemoradiotherapy in locally rectal cancer patients, accurately identifying patients likely to achieve pathological complete response for personalized management. Single‐cell RNA sequencing analysis reveals distinct immune microenvironments underlying model prediction: high‐score tumors are myeloid‐rich and immunosuppressive, whereas low‐score tumors harbor cytotoxic T‐cell‐dominant, elucidating the biological basis of its efficacy.

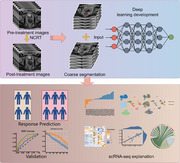

## 1 Introduction

Current clinical guidelines recommend neoadjuvant chemoradiotherapy (NCRT) followed by total mesorectal excision as standard‐of‐care for locally advanced rectal cancer (LARC).^[^ ^1^ ^]^ The achievement of pathological complete response (pCR) following NCRT is observed in up to 30% of patients,^[^ ^2^ ^]^ raising the possibility of organ‐preserving strategies in this subset. This paradigm holds particular clinical significance for some cases where ultra‐low rectal cancer patients prioritizing sphincter preservation, elderly individuals with limited surgery tolerance and younger patients requiring fertility preservation. In such scenarios, the accurate preoperative identification of pCR emerges as a critical need in rectal cancer management.

Magnetic resonance imaging (MRI) has emerged as the cornerstone modality for both primary staging and post‐treatment restaging in rectal cancer.^[^ ^3^ ^]^ The magnetic resonance tumor regression grade (mrTRG), developed by the MERCURY study group, has attracted considerable attention as diagnostic tool to evaluate tumor response.^[^ ^4^, ^5^ ^]^ Previous meta‐analytic study demonstrated mrTRG 1 had suboptimal sensitivity and positive predictive value for predicting pCR,^[^ ^6^ ^]^ which was in accord with results of other studies that have reported over‐staging occurs more frequently than under‐staging on post‐NCRT MRI.^[^ ^7^, ^8^ ^]^ This limitation primarily attribute to challenges in differentiating post‐therapeutic stromal remodeling, characterized by fibrotic transformation and treatment‐related inflammatory infiltrates, from residual viable tumor.

Deep learning provides transformative potential for precision oncology through their capacity to decode complex imaging phenotypes, and has been extensively used in various clinical tasks.^[^ ^9^, ^10^, ^11^, ^12^ ^]^ Historically, prior investigations predominantly focused on static image biomarker from single timepoint during patient care, it failed to capture dynamic pathophysiological evolution during therapy. Emerging studies have evidenced the ability to noninvasively describe tumor phenotypes by integrating longitudinal imaging data was more predictive than that using single timepoint.^[^ ^13^, ^14^, ^15^ ^]^ This paradigm shift underscores the imperative to integrate serial imaging biomarkers into standardized surveillance protocols.

The quest for artificial intelligence‐driven model interpretability has evolved from conventional activation mapping techniques to multimodal frameworks.^[^ ^16^, ^17^ ^]^ Some studies applied deep learning or radiomics to achieve multiscale biological mapping and establish alignments between single‐cell transcriptomic clusters and imaging traits, revealing imaging biomarker mechanisms.^[^ ^18^, ^19^, ^20^ ^]^ This single cell‐resolution interpretability framework not only enhances the model biological validity, but also provides functional bridge between cellular reprogramming events and personalized therapeutic target discovery.

**Figure 1:**
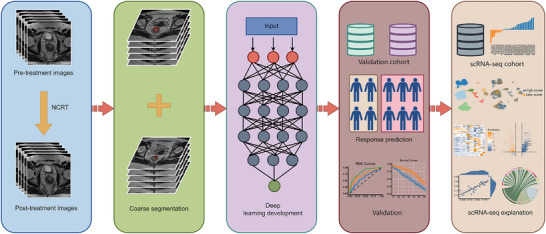
The overall experimental design. The methodological workflow of this study includes the following process: 1) Data acquisition: systematic aggregation and preprocessing of pre‐treatment and post‐treatment MRI scans from locally advanced rectal cancer patients treated with neoadjuvant chemoradiotherapy, establishing imaging inputs for subsequent analysis; 2) Deep learning model development: construction of a deep learning model based on longitudinal MRI for predicting pathological complete response after neoadjuvant chemoradiotherapy; 3) Model evaluation and validation; 4) Explanatory analysis of the model: Establishment of biologically‐grounded causal relationships between model predictions and tumor microenvironment dynamics via single‐cell RNA sequencing analysis, implementing an explainable AI framework. NCRT= neoadjuvant chemoradiotherapy.

Therefore, we aim to develop and validate a deep learning model based on longitudinal MRI for preoperatively pCR prediction in LARC after NCRT (**Figure** **1**). Additionally, to elucidate the molecular mechanisms underlying the model for interpretability through single‐cell RNA sequencing (scRNA‐seq).

## 2 Results

### 2.1 Clinical Characteristics

**Figure 2:**
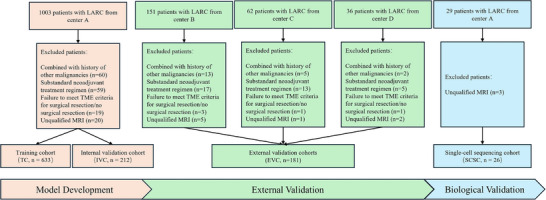
Flowchart of patient inclusion and exclusion. LARC = locally advanced rectal cancer, TME = total mesorectal excision.

A total of 1252 MRI examinations were performed in the four medical centers (**Figure** **2**). The exclusion criteria included patients with other concurrent malignancies (n═80), non‐standardized neoadjuvant treatment regimens (n═94), failure to meet total mesorectal excition criteria for surgical resection or no surgical resection (n═24), and acquired MRI images that were not qualified (n═27). Finally, 1026 patients were enrolled, of whom 633 were assigned to training cohort (TC) (median age, 58 years [IQR, 58‐65]), 212 to the internal validation cohort (IVC) (median age, 55 years [IQR, 55‐64]), and 181 patients to the external validation cohort (EVC) (median age, 56 years [IQR, 56‐62]). The distribution of participant's baseline clinical and pathological information is detailed in **Table** **1**. There were no statistically significant differences in the distribution of baseline clinical information between pCR and non‐pCR patients, except for carcinoembryonic antigen (CEA), distance from inferior part of tumor to the anal verge in the TC and pre‐tumor thickness in the IVC (p═0.01, < 0.001, 0.02, respectively). Above variables with statistically significant differences were incorporated into subsequent multivariate analysis Table S1, Supporting Information).

**Table 1:** Comparison of clinicopathological characteristics between the training, internal and external validation cohorts.

Variable	Training Cohort [TC] [N=633]			Internal Validation Cohort [IVC] [N=212]			External Validation Cohort [EVC] [N=181]			*p*‐method
	pCR (N=104)	Non‐pCR (N=529)	*P value*	pCR (N=35)	Non‐pCR (N=177)	*P value*	pCR (N=31)	Non‐pCR (N=150)	*p value*	
**Age**			0.06			0.39			0.21	Wilcox
Median (Min, Max)	57.0 (27.0, 77.0)	58.0 (22.0, 83.0)		54.0 (27.0, 73.0)	55.0 (22.0, 82.0)		53.0 (29.0, 75.0)	56.0 (14.0, 79.0)		
**Gender**			1.00			0.44			0.20	Chi‐square
Male	33 (31.7%)	167 (31.6%)		13 (37.1%)	51 (28.8%)		12 (38.7%)	38 (25.3%)		
Female	71 (68.3%)	362 (68.4%)		22 (62.9%)	126 (71.2%)		19 (61.3%)	112 (74.7%)		
**CEA**			0.01			0.62			0.26	Chi‐square
Negative	89 (85.6%)	386 (73.0%)		29 (82.9%)	137 (77.4%)		27 (87.1%)	114 (76.0%)		
Positive	15 (14.4%)	143 (27.0%)		6 (17.1%)	40 (22.6%)		4 (12.9%)	36 (24.0%)		
**BMI**			0.78			0.71			0.89	Wilcox
Median (Min, Max)	22.8 (17.7, 31.2)	22.8 (14.5, 35.8)		22.5 (18.4, 31.7)	22.5 (15.9, 30.1)		23.3 (16.7, 28.1)	22.4 (15.0, 298.0)		
**Pre‐T stage**			0.50			0.99			0.76	Chi‐square
T2‐3	67 (64.4%)	319 (60.3%)		22 (62.9%)	108 (61.0%)		15 (48.4%)	80 (53.3%)		
T4	37 (35.6%)	210 (39.7%)		13 (37.1%)	69 (39.0%)		16 (51.6%)	70 (46.7%)		
**Pre‐N stage**			0.99			0.11			0.54	Chi‐square
N0	17 (16.3%)	84 (15.9%)		3 (8.6%)	37 (20.9%)		6 (19.4%)	18 (12.0%)		
N1	37 (35.6%)	192 (36.3%)		16 (45.7%)	54 (30.5%)		9 (29.0%)	46 (30.7%)		
N2	50 (48.1%)	253 (47.8%)		16 (45.7%)	86 (48.6%)		16 (51.6%)	86 (57.3%)		
**Pre‐MRF**			0.70			0.34			0.14	Chi‐square
Negative	79 (76.0%)	414 (78.3%)		29 (82.9%)	130 (73.4%)		26 (83.9%)	103 (68.7%)		
Positive	25 (24.0%)	115 (21.7%)		6 (17.1%)	47 (26.6%)		5 (16.1%)	47 (31.3%)		
**Pre‐EMVI**			0.28			0.30			0.15	Chi‐square
Negative	52 (50.0%)	230 (43.5%)		20 (57.1%)	81 (45.8%)		14 (45.2%)	45 (30.0%)		
Positive	52 (50.0%)	299 (56.5%)		15 (42.9%)	96 (54.2%)		17 (54.8%)	105 (70.0%)		
**Pre‐TIL**			0.14			0.94			0.50	Chi‐square
0.00‐0.50	13 (12.5%)	42 (7.9%)		5 (14.3%)	22 (12.4%)		5 (16.1%)	14 (9.3%)		
0.50‐0.75	38 (36.5%)	168 (31.8%)		10 (28.6%)	54 (30.5%)		8 (25.8%)	37 (24.7%)		
0.75‐1.00	53 (51.0%)	319 (60.3%)		20 (57.1%)	101 (57.1%)		18 (58.1%)	99 (66.0%)		
**Pre‐DTA**			0.01			0.85			0.71	Chi‐square
Low (0‐50)	51 (49.0%)	187 (35.3%)		17 (48.6%)	84 (47.5%)		14 (45.2%)	59 (39.3%)		
Mid (50‐100)	47 (45.2%)	272 (51.4%)		15 (42.9%)	72 (40.7%)		15 (48.4%)	75 (50.0%)		
High (>100)	6 (5.8%)	70 (13.2%)		3 (8.6%)	21 (11.9%)		2 (6.5%)	16 (10.7%)		
**Pre‐Tumor Thickness**			0.88			0.02			0.13	Wilcox
Median (Min, Max)	15.0 (7.0, 116.0)	14.0 (0, 49.0)		13.0 (7.0, 64.0)	15.0 (7.0, 222.0)		15.0 (7.0, 40.0)	17.0 (8.0, 58.0)		

*Note*: Except where indicated, data are numbers of patients, with percentages in parentheses. P values represent the comparison of clinicopathologic variables across all data sets. TIL = Range of tumor involvement in the circumference of intestinal lumen. EMVI = extramural venous invasion. MRF = mesorectal fascia invasion. DTA = Distance from inferior part of tumor to the anal verge. CEA = Carcinoembryonic Antigen. BMI = Body Mass Index.

### 2.2 Performance of MR‐DELTAnet

**Figure 3:**
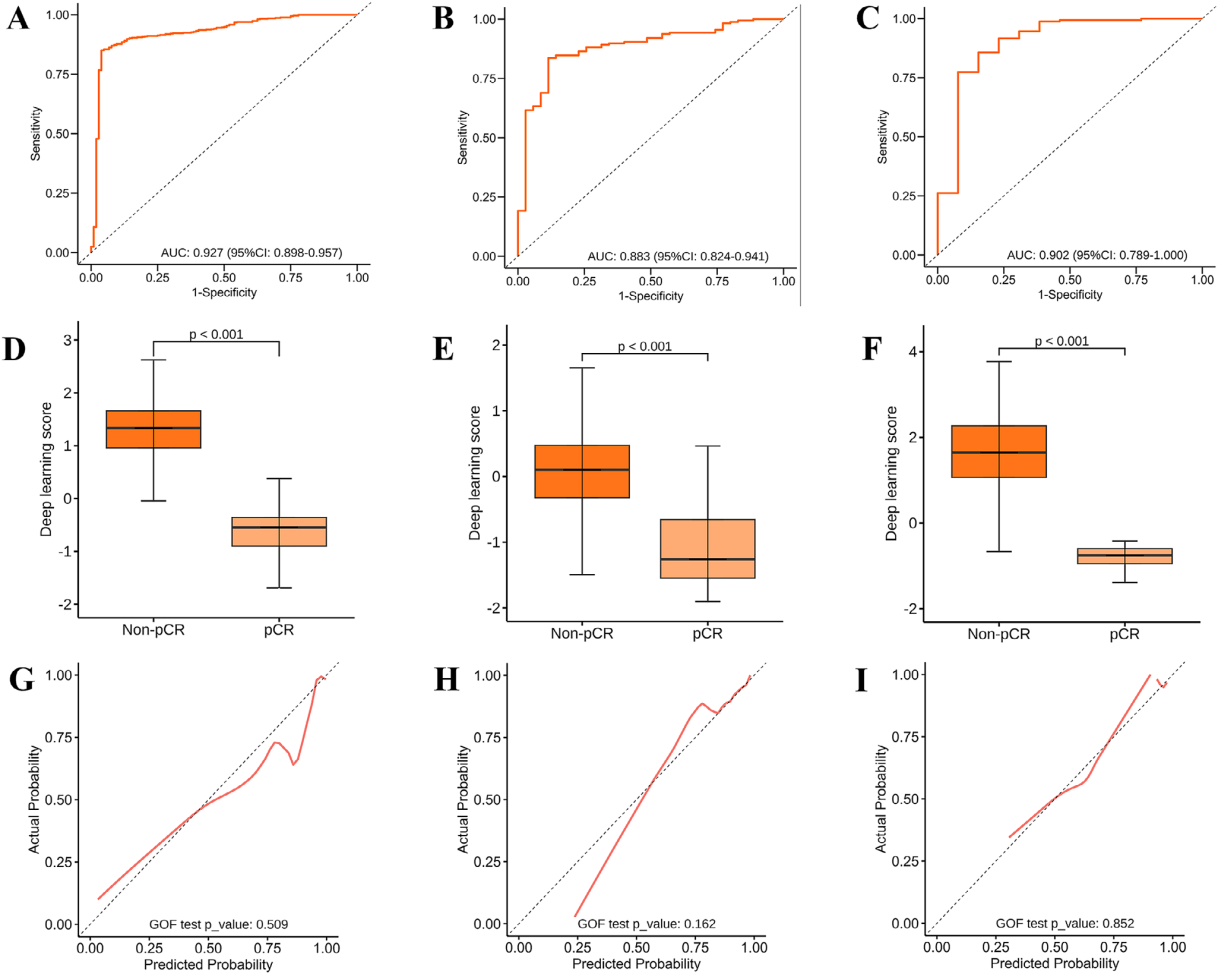
Performance evaluation of the MR‐DELTAnet. ROC curves of the MR‐DELTAnet for the training cohort (A), internal validation cohort (B) and external validation cohort (C). The box plots indicate differences in deep learning score between cohorts according to the MR‐DELTAnet (D‐F). The corresponding calibration curves of the MR‐DELTAnet among cohorts (G–I).

The MR‐DELTAnet achieved an area under the curves (AUC) of 0.93 (95% CI: 0.90, 0.96) in the training cohort. Predictive performance remained robust in validation cohorts, with AUCs of 0.88 (95% CI: 0.82, 0.94) in the internal validation cohort and 0.90 (95% CI: 0.79, 1.00) in the external validation cohort (**Figure** **3**; Table S2, Supporting Information). Ablation studies further confirmed that MR‐DELTAnet consistently demonstrated optimal performance across all datasets (Table S3, Supporting Information). Multivariate analysis identified statistically significant differences in baseline distance from inferior part of tumor to the anal verge (*p* < 0.001) and CEA levels (p═0.01) between pCR and non‐pCR groups. These parameters were subsequently integrated into MR‐DELTAnet to create a fusion model; however, no improvement in predictive performance was observed across any cohort compared to the original model (Table S2, Supporting Information). Guided Grad‐CAM revealed differential localization patterns: for pCR group, activation regions persisted in the tumor core both before and after neoadjuvant chemoradiotherapy; for non‐pCR group, key activation regions concentrated in the tumor core on pre‐treatment T2‐weighted imaging, then shifted peripherally and narrowed after treatment. These results may suggest peripherally invasive tumor spread after treatment in the non‐pCR group, with Guided Grad‐CAM offering information of peritumoral features (Appendix S1 and Figure S1, Supporting Information).

### 2.3 MR‐DELTAnet Associated with Clinical Information and Prognosis

**Figure 4:**
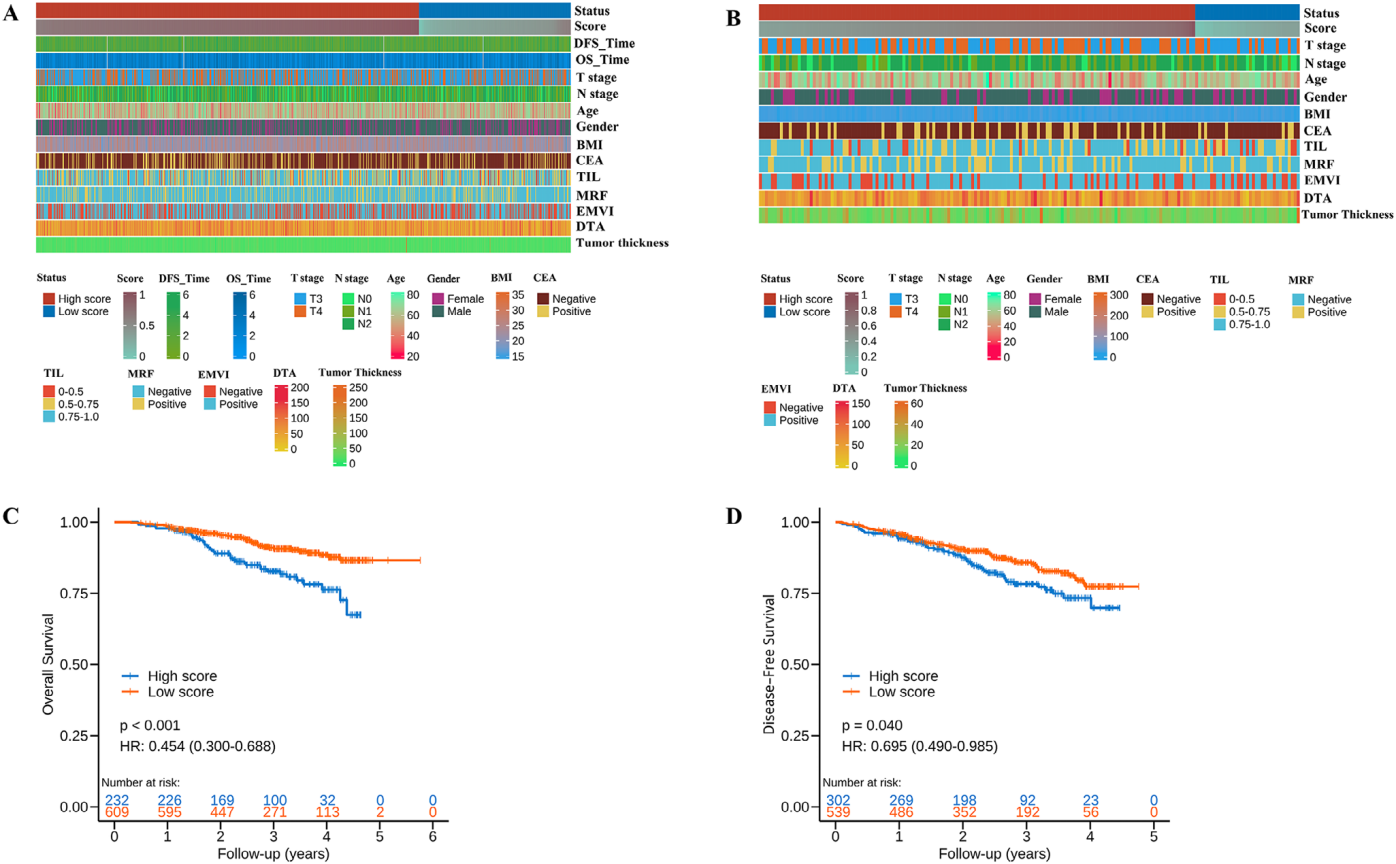
Clinicopathological characteristics and survival outcomes stratified by MR‐DELTAnet prediction scores. Heatmaps illustrate the distribution of clinical characteristics between low‐score and high‐score groups in the development cohort (A) and external validation cohort (B). Kaplan–Meier analysis demonstrates significant differences in disease‐free survival (C) and overall survival (D) between the low‐score and high‐score groups based on the MR‐DELTAnet model. TIL = range of tumor involvement in the circumference of intestinal lumen, EMVI = extramural venous invasion, MRF = mesorectal fascia invasion, DTA = distance from inferior part of tumor to the anal verge, CEA = carcinoembryonic antigen, BMI = body mass index, pCR = pathological complete response.

The displayed integrative heatmap analysis of all patients stratified by model‐predicted response probabilities and clinical characteristics (**Figure** **4**). In the data set from center A, significant differences in baseline lymph node staging were observed, particularly in the distribution of patients with N1 and N2 stages. At the model development center, the baseline distance from inferior part of tumor to the anal verge was greater in the high‐score group (median, 60.0 mm vs 52.0 mm in the low‐score group). In addition, the positive predictive value was lower in the high‐score group. However, these differences were not statistically significant in the external validation dataset. Other variables, such as age, gender and T stage, did not show significant differences in either dataset (Table S4, Supporting Information).

A total of 841 patients who were successfully followed up were included in prognostic analysis, with a median follow‐up of 35 months (IQR, 24.8‐45.2 months) and 30 months (IQR, 18.3‐40.4 months) for overall survival and disease‐free survival, respectively. Kaplan‐Meier analysis showed that the pCR group predicted by the MR‐DELTAnet had significantly better overall survival (OS) and disease‐free survival (DFS) than those predicted to non‐pCR (hazard ratio, 0.51 and 0.70, respectively; both *p* < 0.05, Figure 4). The actual pCR group showed better disease‐free survival compared to the non‐pCR group, but there was no significant statistical difference in overall survival (*p*═0.06, Figure S2, Supporting Information). In terms of clinical characteristics, the univariate analysis revealed that certain parameters, including carcinoembryonic antigen, body mass index (BMI), pre‐treatment T stage (pre‐T stage), pre‐treatment extramural venous invasion (pre‐EMVI) were associated with OS and DFS (Table S5, Supporting Information). Subsequently, in the multivariate Cox regression analysis that incorporated baseline clinical characteristics, the MR‐DELTAnet was identified as an independent predictive factor (Table S6, Supporting Information).

### 2.4 MR‐DELTAnet Associated with Single‐Cell Immune Microenvironment

A total of 26 patients (median age, 52 years; IQR, 43‐64 years) were included in this procedure. Among them, 19 patients (median age, 58 years; IQR, 44.3‐64.8 years) did not achieve complete remission, while 7 patients (median age, 52 years; IQR, 39.3‐52.0 years) achieved pCR. The MR‐DELTAnet was applied to this cohort and demonstrated accurate predictions across the board. Patients were stratified into high‐ and low‐score groups using a threshold of 0.5 according to the model‐based scores (median: 0.66; IQR, 0.49‐0.77).

**Figure 5:**
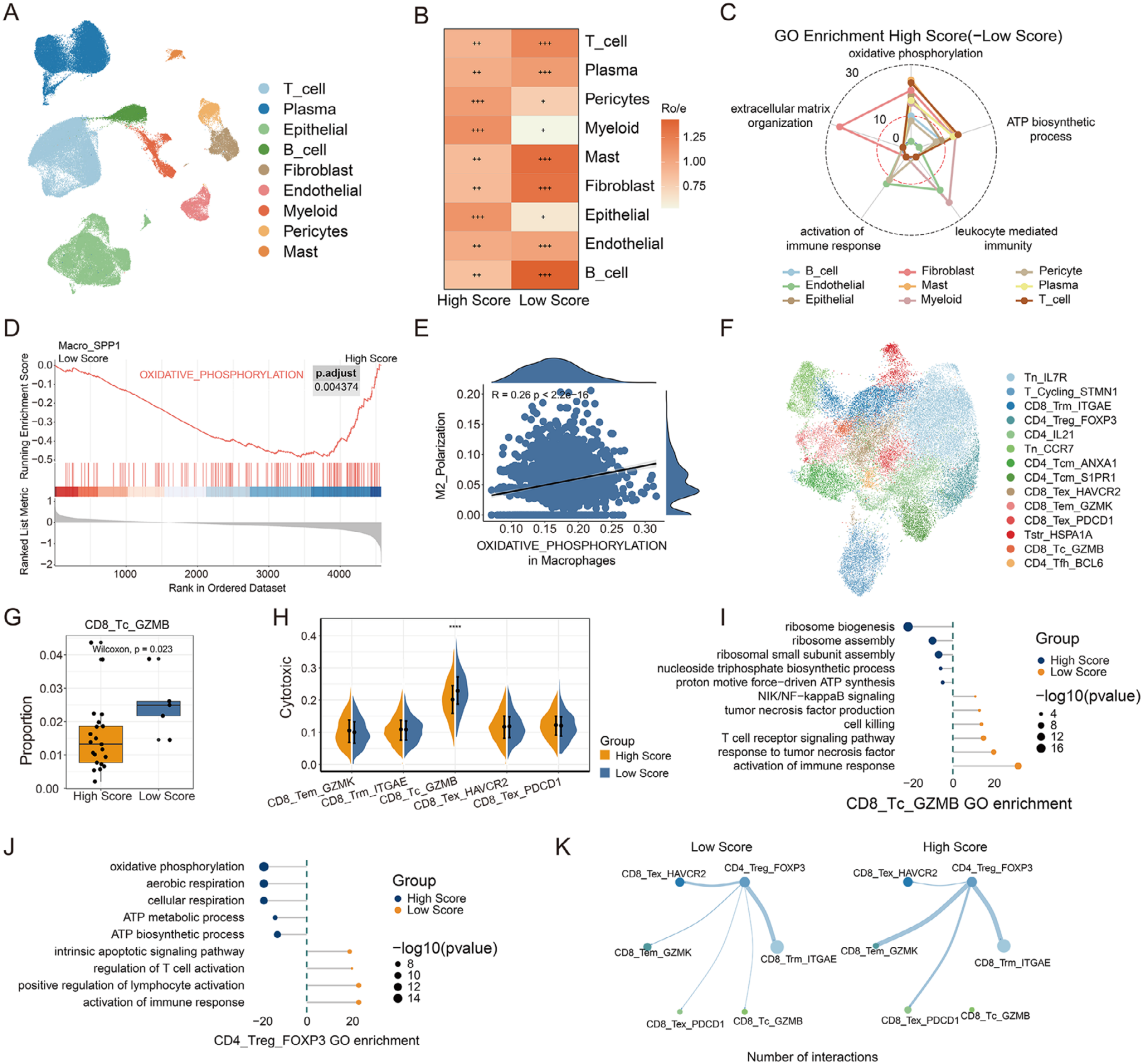
The single cell level immune microenvironment is related to the MR‐DELTAnet model. A) UMAP plot of the major cell types. B) Tissue prevalence of each cell type estimated by Ro/e score, in which Ro/e denotes the ratio of observed to expected cell number. "+++" stands for Ro/e score > 1, "++" stands for Ro/e score ≤ 1 but > 0.8. "+" stands for Ro/e score ≤ 0.8 & ≥ 0.2. "+/−" stands for Ro/e score ≤ 0.2 & > 0. "‐" stands for Ro/e score = 0. C) Radar plot showing the enriched pathways of different cell types in high‐score group (versus low‐score group). The distance from the center point represents the gene count for each pathway. D) GSEA enrichment for oxidative phosphorylation in Macro_SPP1 between high‐ and low‐score groups. E) Dot plot shows the correlation between oxidative phosphorylation score and M2_Polarization score in Macrophage cells. Pearson correlation coefficient. F) UMAP plot of the sub types of T cells. G) Proportion comparison of CD8_Tc_GZMB between high‐ and low‐score groups. Two‐sided Wilcoxon test. H) Gene set scores of cytotoxic among CD8+ T sub types between high‐ and low‐score groups. I) GO enrichment in CD8_Tc_GZMB comparing high‐score versus low‐score. Distance from midline represents the gene count for each pathway. J) GO enrichment in CD4_Treg_FOXP3 comparing High Score versus Low Score. Distance from midline represents the gene count for each pathway. K) Circle plot shows the number of interactions between CD4_Treg_FOXP3 and CD8+ T cells. GO = gene ontology, Ro/e:the ratio of observed over expected cell numbers.

Single‐cell RNA sequencing of 137666 cells from surgical specimens revealed nine major cell types (**Figure** **5**A; Figure S3A, Supporting Information), with distinct transcriptional profiles between high‐ and low‐score groups (Figure S3B,C, Supporting Information). High‐score groups exhibited myeloid cell enrichment (Ro/e analysis), while low‐score groups showed T cell enrichment (Figure 5B). Functional annotation linked high‐score groups to oxidative phosphorylation pathway activation (GO/GSEA; Figure 5C; Figure S3D, Supporting Information), consistent with bulk RNA sequencing data (Figure S3E, Supporting Information). Notably, myeloid cells were further clustered into eight subsets, including macrophage populations (Figure S3F, Supporting Information). Among them, the Macro_SPP1 macrophages in high‐score groups displayed elevated oxidative phosphorylation signatures (Figure S3G, Supporting Information; Figure 5D) and increased M2 polarization scores (Figure S3H, Supporting Information), correlating with immunosuppressive phenotypes (Figure 5E). Then, T cell analysis identified 13 sub‐clusters (Figure 5F; Figure S3I, Supporting Information), with higher CD8_Tc_GZMB proportions in CD8+ T cell subsets (Figure 5G; Figure S3J, Supporting Information) and higher cytotoxic signatures score in low‐score groups (Figure 5H). Differential expression analysis revealed NFKB2, EOMES, KLRT1, and CRTAM upregulation in low‐score groups (Figure S3K, Supporting Information). GO analysis showed immune response activation and NF‐kappa B pathway enrichment in low‐score groups, correlating with cytotoxic scores (Figure 5I; Figure S3L, Supporting Information). In addition, regulatory T cells (Tregs) in high‐score groups showed oxidative phosphorylation pathway enrichment, correlating with inhibitory receptor scores (Figure 5J; Figure S3M, Supporting Information). Cellular communication analysis revealed increased CD4_Treg_FOXP3 signaling in high‐score groups (Figure 5K; Figure S3N, Supporting Information), indicating enhanced immunosuppressive pressure on CD8+ T cells.

## 3 Discussion

This study developed MR‐DELTAnet, an interpretable deep learning model using longitudinal MRI to preoperatively predict pathologic complete response after neoadjuvant chemoradiotherapy in locally advanced rectal cancer, with AUCs of 0.88 and 0.90 in internal and external validation cohorts. The model provided an accurate and non‐invasive tool for preoperative identification of pCR likelihood, thereby potentially informing individualized treatment strategies for LARC management. Single‐cell RNA sequencing of 137666 cells from 26 specimens revealed biological stratification: high‐score group exhibited myeloid enrichment and elevated oxidative phosphorylation, while low‐score group presented cytotoxic CD8+ T cell dominance and enhanced tumor‐killing signatures. These imaging‐biology correlations provide mechanistic support for model‐guided adaptive neoadjuvant therapy strategies.

Recent studies highlight AI‐driven image and clinicopathological biomarkers for superior discrimination accuracy in predicting pCR to neoadjuvant therapy in rectal cancer, outperforming conventional TNM staging.^[^ ^21^, ^22^, ^23^ ^]^ In a prospective study, researchers developed a deep learning model integrating diffusion kurtosis imaging and T2‐weighted imaging to predict pCR following NCRT for LARC, which demonstrated it significantly outperformed subjective assessments by radiologists.^[^ ^24^ ^]^ Cao et al. advanced multi‐sequence MRI analysis using self‐attention mechanism to improve radiomics and deep learning models for therapeutic response prediction.^[^ ^25^ ^]^ Additionally, while deep learning has transformed cancer imaging biomarkers, recent researches shifts prioritize longitudinal monitoring through serial imaging acquisitions, enabling adaptive risk stratification.^[^ ^26^ ^]^ Jin et al. established a basic framework for extracting treatment‐induced change information using longitudinal MRI in rectal cancer, demonstrating improved tumor response prediction with AUCs of 0.95 and 0.92 in two validation cohorts.^[^ ^27^ ^]^ Similarly, in our study, the MR‐DELTAnet achieved AUCs of 0.90 and 0.88 in two validation cohorts by effectively capturing temporal dependencies in longitudinal T2‐weighted images.

This MR‐DELTAnet architecture integrates efficient attention mechanisms to prioritize clinically relevant changes between slices, reducing computational cost while maintaining accuracy. The transformer's inherent ability to model long‐range dependencies enables holistic sequence analysis, which is critical for detecting subtle pathological evolution. In addition, the delta‐efficient design selectively processes significant temporal variation, increasing sensitivity to meaningful biomarkers. By combining temporal attention, computational efficiency and dynamic change modelling, this approach addresses key challenges in sequential medical imaging‐balancing precision with resource constraints. These innovations extend previous methodological benchmarks and demonstrate how optimized temporal feature extraction can advance longitudinal disease characterization and response assessment in oncology imaging.

The tumor microenvironment, a dynamic ecosystem comprising tumor cells, stromal components, immune infiltrates and signaling mediators, critically regulates tumor progression and therapy resistance.^[^ ^28^ ^]^ Neoadjuvant therapy may remodel immunosuppressive tumor microenvironment or activate antitumor immunity. Previously, Feng et al. found that imaging histology model scores correlated with the level of B‐cell infiltration and may be useful for predicting macrotrabecular‐massive hepatocellular carcinoma subtypes, demonstrating non‐invasive insights into the tumor microenvironment.^[^ ^29^ ^]^ However, interpretable MRI deep learning model remains unexplored in rectal cancer. Our MR‐DELTAnet analysis of MRI‐single‐cell integrated data revealed tumor microenvironment heterogeneity: high‐score group exhibited upregulated oxidative phosphorylation across cell types, promoting M2 macrophage polarization and Treg‐mediated immunosuppression, correlating with poor prognosis. Conversely, low‐score group showed enrichment of cytotoxic CD8_Tc_GZMB cells with TNFα pathway activation, indicative of immunostimulatory responses. This study pioneers single‐cell‐level interpretation of imaging‐based tumor microenvironment stratification in rectal cancer, explaining how radiomic features reflect immune‐modulating mechanisms. By associating deep learning signatures with immunosuppressive or immunogenic microenvironments, our model provides non‐invasive biomarkers for therapeutic response prediction and mechanistic insights into resistance, advancing precision oncology in neoadjuvant settings.

The MR‐DELTAnet demonstrated superior prognostic stratification potency in overall survival compared to conventional histopathological assessment using tumor regression grade criteria. This divergence likely stems from fundamental differences in biological interpretability. Current tumor regression grade protocols predominantly focus on residual tumor quantification, while the MR‐DELTAnet integrated longitudinal MRI to map spatial heterogeneity and decode tumor microenvironment dynamics, which was validated by single‐cell RNA sequencing analysis. In light of these findings, deep learning based longitudinal imaging may emerge as an effective strategy to capture spatiotemporal tumor evolution during therapy, enabling precise treatment response monitoring and prognostic prediction.

Although our study has provided valuable insights, there are several limitations that should be acknowledged. First, it is retrospective study and subject to potential selection bias, prospective studies in future are warranted to test the generalizability and clinical utility of the proposed model. Additionally, the sample size included in the scRNA‐seq analysis is relatively limited, which may constrain its ability to comprehensively identify and characterize rare cell subpopulations within the tissue, and might also affect the generalizability of the findings. Future studies could address this constraint by expanding the sample size through multi‐center collaborations. Second, manual ROI delineation on pre‐ and post‐treatment MRIs is a limitation, given its labor intensity and lack of reproducibility metrics. It was adopted owing to substantial anatomical variation from peristalsis and lesion regression after therapy, which hinder reliable automated segmentation. Nonetheless, this expert‐supervised process ensured high‐quality annotations that may support future development of deep learning–based segmentation frameworks such as nnU‐Net. Third, conventional rectal MRI protocols are inherently multiparametric, but this study focused exclusively on T2‐weighted imaging, which inevitably neglects complementary biological information encoded in other sequences and may introduce analysis bias. Despite the MR‐DELTAnet based longitudinal T2‐weighted imaging demonstrated outstanding performance in predicting pCR and presented robustness across validation cohorts, future iterations of this framework would benefit from integrating multiparametric MRI data.

In conclusion, MR‐DELTAnet, a deep learning framework integrating longitudinal MRI, enables noninvasive prediction of pathological complete response to neoadjuvant chemoradiotherapy in locally advanced rectal cancer, while revealing distinct immune infiltration landscapes underlying its stratification. The model's ability to stratify patients based on differential immune cell compositions and pathway activation patterns underscores its clinical relevance for optimizing precision treatment strategies in rectal cancer management.

## 4 Experimental Section

### Study Design and Participants

This study was conducted in accordance with the ethical principles of the Declaration of Helsinki and received approval from the Institutional Review Board (IRB) of the Sixth Affiliated Hospital, Sun Yat‐sen University (Approval No. 2024ZSLYEC‐702). Due to the retrospective nature of the study, the requirement for informed consent was waived by the ethics committee. All patient data were anonymized and de‐identified prior to analysis to protect privacy and confidentiality. Data access and usage complied with institutional data protection policies and relevant national regulations.

We enrolled adults (≥ 18 years) with pathologically confirmed LARC (cT3‐4N_any_ or cT2N+), excluding those with distant metastases. Inclusion criteria included: 1) pre‐/post‐NCRT MRI, 2) completed NCRT followed by total mesorectal excision, and 3) available pathology reports. Exclusion criteria included: prior rectal cancer treatment or incomplete treatment, major comorbidities (cardiac, pulmonary, hepatic or renal dysfunction), concurrent malignancies (except non‐melanoma skin cancer), MRI contraindications or poor image quality. The standardized selection process (Figure 2; Appendix S2, Supporting Information) ensured cohort homogeneity for longitudinal MRI‐based treatment response evaluation.

We retrospectively enrolled 845 LARC patients from center A (July 2012‐July 2023), randomly divided them into training cohort (TC, n═633) and internal validation cohort (IVC, n═212) with a ratio of 0.75: 0.25. An external validation cohort (EVC, n═181) comprised patients from three additional centers (center B: n═113, center C: n═42, center D: n═26). Additionally, between January 2021 and September 2021, the single‐cell RNA sequencing data from 26 patients at Center A, collected and analyzed in the previous study,^[^ ^30^ ^]^ formed a dedicated validation cohort (SCSC) for immune microenvironment analysis. We collected clinical data including age, gender, baseline clinical TNM staging, status of pre‐treatment range of tumor involvement in the circumference of intestinal lumen, mesorectal fasciae invasion and extramural vascular invasion, as well as the tumor thickness and the baseline distance from inferior part of tumor to the anal verge. The neoadjuvant chemoradiotherapy regimens of patients were shown in Appendix S3 (Supporting Information). Furthermore, the follow‐up data was collected for survival analysis (Appendix S4, Supporting Information).

### Histopathologic Assessment and Definition of pCR

The pathological T and N stages after NCRT were evaluated according to the American Joint Committee on Cancer.^[^ ^31^ ^]^ The pCR was defined as absence of viable tumor cell within the resected primary tumor specimen and all sampled regional lymph nodes (Appendix S5, Supporting Information).

### MRI Data Acquisition and Image Preprocessing

MRI acquisition parameters and image preprocessing are shown in Appendix S6 and Table S7 (Supporting Information). Tumor regions of interest (ROIs) were manually segmented on axial T2‐weighted images using ITK‐SNAP (v2.2.0). While pre‐treatment tumour boundaries were readily identifiable, post‐treatment ROIs required slice‐wise comparison with baseline. ROIs were drawn to encompass the entire original tumour site, including residual disease or treatment‐related fibrosis, excluding adjacent bowel lumen, air and normal tissue. To ensure accurate localization, additional sequences may be incorporated, and a comprehensive preprocessing pipeline was implemented to address data variability arising from multicenter instrumentation and parameter differences (Appendix S6, Supporting Information).

### MR‐DELTAnet Development and Validation

**Figure 6:**
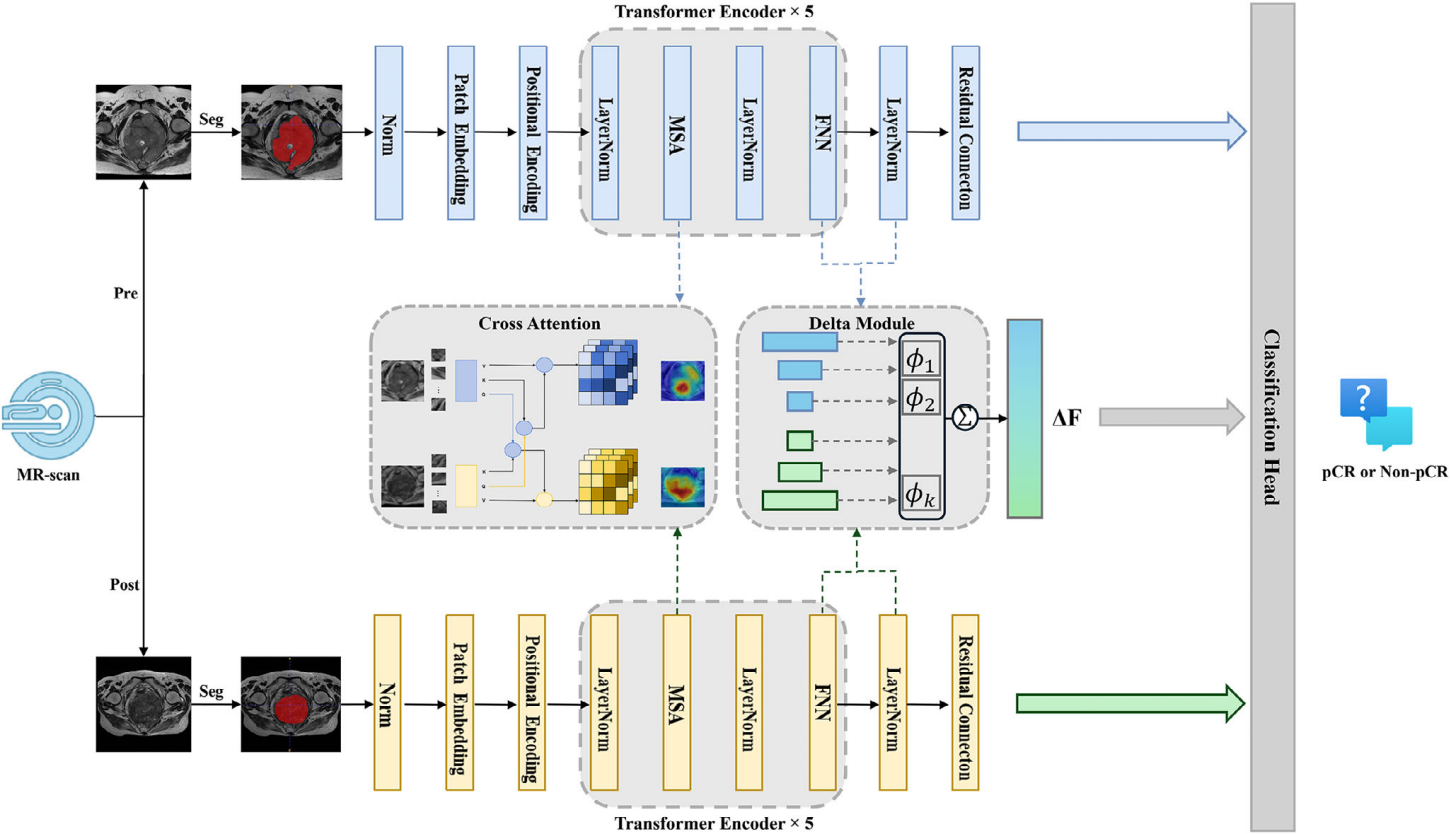
Flowchart of MR‐DELTAnet. The model integrates Transformer blocks with a cross‐attention mechanism and a Delta module designed to extract multi‐scale feature differences, capturing dynamic changes between pre‐ and post‐treatment T2‐weighted images, and outputs results through a classification decision module; it also employs a dual optimization strategy of dynamic data augmentation and balanced batch training to effectively address sample imbalance among treatment response categories. pCR = pathological complete response, MSA = Multi‐Head Self‐Attention, FFN = Feed‐Forward Network.

We developed MR‐DELTAnet, a Transformer‐based medical image analysis model, to predict treatment response using pre‐ and post‐treatment MR images (**Figure** **6**; Appendix S7, Supporting Information). The architecture integrates three core components: 1) a cross‐attention mechanism aligns pre‐ and post‐treatment image features by computing correlation matrices, directing focus to regions with significant treatment‐related changes; 2) a Delta module that quantifies multi‐scale feature disparities across time points to capture dynamic treatment effects; 3) a Classification Decision Module for final predictions. We addressed class imbalance via dynamic data augmentation and balanced batch sampling, coupled with an adaptive loss function combining Class‐Balanced Focal Loss and Cross‐time Contrastive Regularization to enhance robustness. The model was trained using AdamW optimization with stratified learning rates, Stochastic Depth regularization, and F1‐score‐based early stopping on a supercomputing cluster. Ablation studies systematically validated the contribution of each component to pCR prediction (Appendix S8, Supporting Information). Our code is available at https://github.com/WuLei‐MedIA/mr_deltanet.

The predictive performance of MR‐DELTAnet was evaluated using receiver operating characteristic (ROC) curves, with area under the curve (AUC), sensitivity, specificity and accuracy calculated at the optimal threshold determined by the Youden index. Calibration curves assessed the agreement between predicted probabilities and observed pCR outcomes.

### Interpretation and Molecular Validation of the Model

Model interpretability was enhanced by Guided Gradient‐weighted Class Activation Mapping (Guided Grad‐CAM) visualization to identify imaging regions contributing to predictions (Appendix S1, Supporting Information). Spearman's rank correlation analysis was used to explore associations between model prediction and single‐cell RNA sequencing‐derived transcriptional profile. To characterize the tumour microenvironment, patients were stratified into subgroups using unsupervised clustering of MR‐DELTAnet‐derived deep features. Between‐group differences in immune infiltration, stromal composition and intercellular communication networks were assessed using the Mann‐Whitney U test or Fisher's exact test The Single‐cell RNA Sequencing Workflow was presented in Appendix S9 (Supporting Information).

### Statistical Analysis

Continuous and categorical variables were presented as medians with interquartile ranges (IQRs) and frequencies with percentages. Patient characteristics in each cohort were compared using the Fisher exact test or Pearson's χ^2^ test for categorical variables and the Kruskal‐Wallis test for continuous variables. Univariable and multivariable logistic regression analyses were performed to assess the relationship between patient characteristics and the likelihood of achieving pCR, with results reported as odds ratios (ORs) and 95% confidence intervals (CIs). The Kaplan‐Meier was utilized to evaluate prognosis by model‐based scores and pathological findings, with overall survival (OS) and disease‐free survival (DFS) serving as primary evaluation metrics. Additionally, univariate and multivariate Cox regression analyses were performed to evaluate the associations of the baseline clinical characteristics and the model prediction scores with survival outcomes. All analyses were performed using R software (version 3.6.0), with two‐tailed p values < 0.05 considered statistically significant.

### Ethics Approval and Patient Consent Statement

This study was conducted in accordance with the ethical principles of the Declaration of Helsinki and received approval from the Institutional Review Board (IRB) of The Sixth Affiliated Hospital, Sun Yat‐sen University (Approval No. 2024ZSLYEC‐702). Due to the retrospective nature of the study, the requirement for informed consent was waived by the ethics committee.

## Conflict of Interest

The authors declare no conflict of interest.

## Supporting information



Supporting Information

## Data Availability

The data that support the findings of this study are available from the corresponding author upon reasonable request.
