# Supplementary material for: MR‐DELTAnet: A Longitudinal MRI‐Transformer Model Predicting Pathological Complete Response and Revealing Immune Microenvironment via scRNA‐seq in Locally Advanced Rectal Cancer
Source: Adv Sci (Weinh). 2025 Dec 19;13(11):e17721. doi: 10.1002/advs.202517721 (PMC12931229; doi:10.1002/advs.202517721)
Supplement: Supplementary file 1 — Supporting Information [file ADVS-13-e17721-s001.docx]

**Supplementary Tables**

**Table S1. Multifactorial analysis based on baseline clinical characteristics**

|  |  | Coefficient | OR (95%CI) | *P* Value |
| --- | --- | --- | --- | --- |
| Intercept |  | -0.875 | 0.417 (0.218, 0.796) | 0.007 |
| CEA |  |  |  |  |
| Negative |  | reference | —— | —— |
| Positive |  | -0.789 | 0.454 (0.245, 0.793) | 0.008 |
| Pre-DTA |  | -0.014 | 0.986 (0.798, 0.994) | 0.0004 |
| Pre-Tumor Thickness |  | 0.014 | 1.014 (0.986, 1.043) | 0.291 |

Note. CEA = carcinoembryonic Antigen. DTA = distance from inferior part of tumor to the anal verge.

**Table S2. Comparison of the predictive efficiency of clinical features, MR-DELTAnet models and their fusion**

| **Dataset** | **Model** | **AUC**  **(95%CI)** | **Accuracy**  **(95%CI)** | **Sensitivity**  **(95%CI)** | **Specificity**  **(95%CI)** | **PPV**  **(95%CI)** | **NPV**  **(95%CI)** |
| --- | --- | --- | --- | --- | --- | --- | --- |
| Training Cohort  (TC) | Clinical | 0.632  (0.576,0.687) | 0.408  (0.407,0.408) | 0.310  (0.271,0.349) | 0.904  (0.847,0.961) | 0.943  (0.908,0.977) | 0.205  (0.168,0.242) |
|  | MR-DELTAnet | 0.927  (0.898,0.957) | 0.869  (0.869,0.869) | 0.851  (0.820,0.881) | 0.962  (0.925,0.998) | 0.991  (0.983,1.000) | 0.559  (0.486,0.631) |
|  | MR-DELTAnet +clinical | 0.937  (0.911,0.963) | 0.874  (0.873,0.874) | 0.858  (0.828,0.888) | 0.952  (0.911,0.993) | 0.989  (0.980,0.999) | 0.569  (0.495,0.643) |
| Internal Validation Cohort  (IVC) | Clinical | 0.529  (0.424,0.634) | 0.292  (0.291,0.294) | 0.164  (0.109,0.218) | 0.943  (0.866,1.000) | 0.935  (0.871,1.000) | 0.182  (0.126,0.239) |
|  | MR-DELTAnet | 0.883  (0.824,0.941) | 0.844  (0.843,0.846) | 0.836  (0.782,0.891) | 0.886  (0.780,0.991) | 0.974  (0.948,0.999) | 0.517  (0.390,0.643) |
|  | MR-DELTAnet + Clinical | 0.882  (0.827,0.938) | 0.840  (0.838,0.841) | 0.831  (0.775,0.886) | 0.886  (0.780,0.991) | 0.974  (0.948,0.999) | 0.508  (0.383,0.634) |
| External Validation Cohort  (EVC) | Clinical | 0.549  (0.400,0.699) | 0.597  (0.594,0.599) | 0.595  (0.521,0.669) | 0.615  (0.351,0.880) | 0.952  (0.912,0.993) | 0.105  (0.036,0.174) |
|  | MR-DELTAnet | 0.902  (0.789,1.000) | 0.856  (0.855,0.858) | 0.857  (0.804,0.910) | 0.846  (0.650,1.000) | 0.986  (0.967,1.000) | 0.314  (0.160,0.468) |
|  | MR-DELTAnet + Clinical | 0.903  (0.800,1.000) | 0.917  (0.916,0.918) | 0.929  (0.890,0.968) | 0.769  (0.540,0.998) | 0.981  (0.962,1.000) | 0.455  (0.246,0.663) |

Note. AUC = the area under the ROC curve, NPV = negative predictive value, PPV = positive predictive value.

**Table S3. Comparison of the ablation experiments**

| **Dataset** | **Pre** | **Post** | **Cross-Attention** | **Delta**  **Module** | **Training Set AUC (95%CI)** | **Internal Validation Set AUC (95%CI)** | **External Validation AUC (95%CI)** |
| --- | --- | --- | --- | --- | --- | --- | --- |
| VGG | **√** | **√** |  |  | 0.808  (0.761, 0.856) | 0.791  (0.703, 0.880) | 0.774  (0.615, 0.933) |
| ResNet | **√** | **√** |  |  | 0.852  (0.804, 0.899) | 0.839  (0.753, 0.924) | 0.802  (0.664, 0.939) |
| Transformer | **√** |  |  |  | 0.831  (0.786, 0.875) | 0.801  (0.720, 0.882) | 0.763  (0.613, 0.913) |
|  |  | **√** |  |  | 0.840  (0.797, 0.884) | 0.811  (0.724, 0.897) | 0.789  (0.686, 0.892) |
|  | **√** | **√** | **√** |  | 0.878  (0.845, 0.910) | 0.837  (0.751, 0.922) | 0.802  (0.651, 0.953) |
| MR-DELTAnet | **√** | **√** | **√** | **√** | 0.927  (0.898,0.957) | 0.883  (0.824,0.941) | 0.902  (0.789,1.000) |

Note. AUC = the area under the ROC curve

**Table S4. Patient characteristics of model subgroups in different centres**

| **Variable** | **MR-DELTAnet**  **(Center A)**  **（N=845）** | | |  | | **MR-DELTAnet**  **(External Validation Centers)**  **（N=181）** | | | ***P*-method** |
| --- | --- | --- | --- | --- | --- | --- | --- | --- | --- |
|  | **Low-score Group**  **(N=218)** | **High-score Group**  **(N=627)** | ***P*** | |  | **Low-score Group**  **(N=46)** | **High-score Group**  **(N= 135)** | ***P*** |  |
| **Age** |  |  | 0.51919 | |  |  |  | 0.52392 | Mann-Whitney U |
| Median (Min, Max) | 57.0  (27.0, 77.0) | 58.0  (22.0, 83.0) |  | |  | 55.0  (29.0, 79.0) | 56.0  (14.0, 79.0) |  |  |
| **Gender** |  |  | 0.45632 | |  |  |  | 0.493645 | Chi-square |
| Male | 145 (66.5%) | 436 (31.6%) |  | |  | 31 (67.4%) | 100 (74.1%) |  |  |
| Female | 73 (33.5%) | 191 (68.4%) |  |  |  | 15 (32.6%) | 35 (25.9%) |  |  |
| **CEA** |  |  | 0.09347 | |  |  |  | 0.784138 | Chi-square |
| Negative | 175 (80.3%) | 161 (25.7%) |  | |  | 37 (80.4%) | 104 (77.0%) |  |  |
| Positive | 43 (19.7%) | 446 (74.3%) |  | |  | 9 (19.6%) | 31 (23.0%) |  |  |
| **BMI** |  |  | 0.68848 | |  |  |  | 0.81957 | Mann-Whitney U |
| Median (Min, Max) | 22.7  (16.3, 31.7) | 22.8  (14.5, 35.8) |  | |  | 22.775  (15.8, 28.1) | 22.3  (15.0, 36.0) |  |  |
| **Pre-T stage** |  |  | 0.58595 | |  |  |  | 0.36611 | Chi-square |
| T2-3 | 137 (62.8%) | 379 (60.5%) |  | |  | 21 (45.7%) | 74 (54.9%) |  |  |
| T4 | 81 (37.2%) | 248 (39.5%) |  | |  | 25 (54.3%) | 61 (45.2%) |  |  |
| **Pre-N stage** |  |  | ＜0.0001 | |  |  |  | 0.840657 | Chi-square |
| N0 | 31 (14.2%) | 110 (17.5%) |  | |  | 6 (13.0%) | 18 (13.3%) |  |  |
| N1 | 165 (75.7%) | 512 (81.7%) |  | |  | 40 (87.0%) | 116 (85.9%) |  |  |
| N2 | 22 (10.1%) | 2 (0.8%) |  | |  | 0 (0%) | 1 (0.8%) |  |  |
| **Pre-MRF** |  |  | 0.48735 | |  |  |  | 1 | Chi-square |
| Negative | 164 (75.2%) | 488 (77.8%) |  | |  | 33 (71.7%) | 96 (71.1%) |  |  |
| Positive | 54 (24.8%) | 139 (22.2%) |  | |  | 13 (28.3%) | 39 (28.9%) |  |  |
| **Pre-EMVI** |  |  | 0.45879 | |  |  |  | 0.201677 | Chi-square |
| Negative | 104 (47.7%) | 279 (44.5%) |  | |  | 19 (41.3%) | 40 (29.6%) |  |  |
| Positive | 114 (52.3%) | 348 (55.5%) |  | |  | 27 (58.7%) | 95 (70.4%) |  |  |
| **Pre-TIL** |  |  | 0.568375 | |  |  |  | 0.983316 | Chi-square |
| 0.00-0.50 | 25 (11.5%) | 57 (9.1%) |  | |  | 5 (10.9%) | 14 (10.4%) |  |  |
| 0.50-0.75 | 70 (32.1%) | 200 (31.9%) |  | |  | 11 (23.9%) | 34 (25.2%) |  |  |
| 0.75-1.00 | 123 (56.4%) | 370 (59.0%) |  | |  | 30 (65.2%) | 87 (64.4%) |  |  |
| **PRE-DTA** |  |  | 0.01701 | |  |  |  | 0.779 | Mann-Whitney U |
| Median (Min, Max) | 52.0  (0, 155.0) | 60.0  (0, 159.0) |  | |  | 56.0  (0.0, 117.0) | 55.0  (5.0, 147.0) |  |  |
| **Pre-Tumor Thickness** |  |  | 0.446377 | |  |  |  | 0.18372 | Mann-Whitney U |
| Median (Min, Max) | 14.0  (7.0, 116.0) | 14.0  (0, 222.0) |  | |  | 15.0  (7.0, 40.0) | 17.0  (9.0, 58.0) |  |  |
| **Pre-Tumor Deposit** |  |  | 0.03574 | |  |  |  | 0.989201 | Chi-square |
| Negative | 209 (95.9%) | 618 (98.6%) |  | |  | 46 (100%) | 133 (98.5%) |  |  |
| Positive | 9 (4.1%) | 9 (1.4%) |  | |  | 0 (8.6%) | 2 (1.5%) |  |  |
| **Pre-Longitudinal Diameter** |  |  | 0.834213 | |  |  |  | 0.470266 | Mann-Whitney U |
| Median (Min, Max) | 43.0 | 43 |  | |  | 50 | 47.0 |  |  |
|  | (15.0, 90.0) | (0.0, 488.0) |  | |  | (19.0, 87.0) | (15.0, 122.0) |  |  |

Note. Except where indicated, data are numbers of patients, with percentages in parentheses. P values represent the comparison of clinicopathologic variables across all data sets. TIL = range of tumor involvement in the circumference of intestinal lumen. EMVI = extramural venous invasion. MRF = mesorectal fascia invasion. DTA = distance from inferior part of tumor to the anal verge. CEA = carcinoembryonic antigen. BMI = body mass index.

**Table S5. Univariate analysis of baseline clinical characteristics and the MR-DELTAnet score for survival outcomes.**

| **Variables** | **Overall Survival** | |  | **Disease-free Survival** | |
| --- | --- | --- | --- | --- | --- |
|  | **HR (95% CI)** | **p Value** |  | **HR (95% CI)** | **p Value** |
| Gender (Male vs. Female) | 1.168 (0.740-1.842) | 0.505 |  | 0.969 (0.670-1.401) | 0.867 |
| Age | 1.022 (1.002-1.042) | 0.030 |  | 1.014 (0.998-1.031) | 0.082 |
| CEA (Negative vs. Positive) | 2.933 (1.936-4.443) | ＜0.001 |  | 2.653 (1.869-3.767) | ＜0.001 |
| BMI | 0.828 (0.765-0.895) | 0.0000023 |  | 0.893 (0.838-0.952) | 0.0005084 |
| Pre-T stage (T2-3 vs. T4) | 3.905 (2.498-6.103) | ＜0.001 |  | 2.972 (2.077-4.252) | ＜0.001 |
| Pre-N stage (N0 vs. N1) | 0.866 (0.462-1.620) | 0.652 |  | 1.027 (0.588-1.796) | 0.925 |
| Pre-N stage (N0 vs. N2) | 1.085 (0.607-1.941) | 0.783 |  | 1.398 (0.833-2.345) | 0.204 |
| Pre-MRF (Negative vs. Positive) | 1.075 (0.664-1.739) | 0.769 |  | 1.135 (0.761-1.693) | 0.536 |
| Pre-EMVI (Negative vs. Positive) | 2.835 (1.752-4.586) | ＜0.001 |  | 2.574 (1.738-3.812) | ＜0.001 |
| Pre-TIL (0.50-0.75 vs. 0.00-0.50) | 0.696 (0.317-1.528) | 0.366 |  | 0.923 (0.450-1.894) | 0.826 |
| Pre-TIL (0.75-1.00 vs. 0.00-0.50) | 1.194 (0.593-2.405) | 0.620 |  | 1.629 (0.847-3.134) | 0.143 |
| Pre-DTA (Mid vs. Low) | 0.901 (0.566-1.434) | 0.659 |  | 0.997 (0.683-1.455) | 0.988 |
| Pre-DTA (High vs. Low) | 1.802 (1.030-3.151) | 0.039 |  | 1.273 (0.758-2.139) | 0.362 |
| Pre-Tumor Thickness | 0.997 (0.973-1.023) | 0.836 |  | 0.997 (0.977-1.018) | 0.805 |
| MR-DELTAnet score | 0.454 (0.300-0.688) | 0.0002003 |  | 0.695 (0.490-0.985) | 0.041 |

Note. CEA = carcinoembryonic antigen. BMI = body mass index. MRF = mesorectal fascia invasion. EMVI = extramural venous invasion. TIL = range of tumor involvement in the circumference of intestinal lumen. DTA = distance from inferior part of tumor to the anal verge.

**Table S6. Multivariate analysis of baseline clinical characteristics and the MR-DELTAnet score for survival outcomes.**

| **Variables** | **Overall Survival** | |  | **Disease-free Survival** | |
| --- | --- | --- | --- | --- | --- |
|  | **HR (95% CI)** | **p Value** |  | **HR (95% CI)** | **p Value** |
| CEA (Negative vs. Positive) | 2.243 (1.573-3.199) | ＜0.001 |  | 2.317 (1.628-3.296) | ＜0.001 |
| BMI | 0.919 (0.859-0.983) | 0.014 |  | 0.922 (0.862-0.985) | 0.017 |
| Pre-T stage (T2-3 vs. T4) | 2.309 (1.587-3.358) | ＜0.001 |  | 2.397 (1.648-3.487) | ＜0.001 |
| Pre-EMVI (Negative vs. Positive) | 1.752 (1.161-2.644) | 0.008 |  | 1.787 (1.184-2.696) | 0.008 |
| MR-DELTAnet score | 0.655 (0.456-0.940) | 0.021 |  | 0.660 (0.464-0.938） | 0.020 |

Note. CEA = carcinoembryonic antigen. BMI = body mass index. EMVI = extramural venous invasion.

**Table S7. MRI scanning parameters for different centers**

| Center | Scanner | Sequence | Parameters | | | | | |
| --- | --- | --- | --- | --- | --- | --- | --- | --- |
|  |  |  | TR (ms) | TE (ms) | FOV (mm) | Matrix | NEX | Section Thickness  /Gap (mm) |
| Center A | GE, MR 750  3.0T | Sagittal T2WI | 3500 | 110 | 27 | 288/288 | 2 | 3.0/0.3 |
|  |  | Oblique Axial T2WI | 5694 | 110 | 18 | 288/256 | 4 | 3.0/0.3 |
|  |  | Oblique Coronal T2WI | 5000 | 110 | 18 | 288/256 | 4 | 3.0/0.3 |
|  |  | Oblique Axial DWI (b = 1000 s/mm2) | 2800 | 60 | 34 | 128/128 | 6 | 4.0/0.5 |
|  |  | Oblique Axial T1WI | 560 | 9.1 | 22 | 288/256 | 2 | 3.0/0.3 |
|  | GE, Optima MR 360  1.5T | Sagittal T2WI | 2591 | 125 | 40 | 288/224 | 4 | 3.0/0.5 |
|  |  | Oblique Axial T2WI | 4294 | 108 | 24 | 288/256 | 4 | 3.0/0.5 |
|  |  | Oblique Coronal T2WI | 2358 | 108 | 24 | 288/256 | 4 | 3.0/0.5 |
|  |  | Oblique Axial DWI (b = 1000 s/mm2) | 4550 | 92.6 | 40 | 192/192 | 4 | 3.0/0.5 |
|  |  | Oblique Axial T1WI | 419 | 13.5 | 24 | 320/224 | 2 | 3.0/0.5 |
| Center B | GE, Signa Hdi  1.5T | Sagittal T2WI | 3080 | 110 | 26 | 288/224 | 2 | 4.0/1.0 |
|  |  | Oblique Axial T2WI | 4440 | 112 | 28 | 384/224 | 2 | 4.0/0.8 |
|  |  | Oblique Coronal T2WI | 4500 | 102 | 25.6 | 200/200 | 2 | 4.0/1.0 |
|  |  | Oblique Axial DWI (b = 1000 s/mm2) | 4675 | 79.9 | 28 | 128/128 | 4 | 4.0/1.0 |
|  |  | Oblique Axial T1WI | 205 | 4.2 | 35 | 256/224 | 2 | 4.0/1.0 |
|  | Philips, Prodiva  1.5T | Sagittal T2WI | 2000 | 100 | 18 | 276/200 | 1 | 4.0/1.0 |
|  |  | Oblique Axial T2WI | 2000 | 100 | 18 | 256/207 | 1.4 | 3.0/0 |
|  |  | Oblique Coronal T2WI | 2000 | 100 | 18 | 258/209 | 1.2 | 3.0/0 |
|  |  | Oblique Axial DWI (b = 1000 s/mm2) | 3000 | 65 | 22 | 72/74 | 4 | 4.0/1.0 |
|  |  | Oblique Axial T1WI | 445 | 10 | 18 | 240/211 | 1.6 | 3.0/0 |
| Center C | GE, Architect  3.0T | Sagittal T2WI | 3518 | 110 | 24 | 320/256 | 2 | 3.0/0.5 |
|  |  | Oblique Axial T2WI | 3518 | 110 | 24 | 384/384 | 2 | 3.0/0.5 |
|  |  | Oblique Coronal T2WI | 3872 | 110 | 24 | 320/320 | 2 | 3.0/0.5 |
|  |  | Oblique Axial DWI (b = 1000 s/mm2) | 5800 | 75 | 24 | 150/152 | 4 | 3.0/0.5 |
|  |  | Oblique Axial T1WI | 423 | 17 | 24 | 150/152 | 2 | 3.0/0.5 |
|  | Simens, Magnetom Aera  1.5T | Sagittal T2WI | 2700 | 78 | 23 | 256/300 | 2 | 4.0/0.8 |
|  |  | Oblique Axial T2WI | 2400 | 84 | 20 | 266/320 | 2 | 4.0/0.8 |
|  |  | Oblique Coronal T2WI | 2800 | 80 | 22 | 240/320 | 2 | 4.0/0.8 |
|  |  | Oblique Axial DWI (b = 1000 s/mm2) | 2800 | 94 | 23 | 106/176 | 4 | 4.0/0.8 |
|  |  | Oblique Axial T1WI | 200 | 200 | 20 | 218/256 | 2 | 4.0/0.8 |
| Center D | GE, SIGNA premier  3.0T | Sagittal T2WI | 3895 | 102 | 24 | 320/320 | 1.5 | 4.0/0.4 |
|  |  | Oblique Axial T2WI | 3910 | 102 | 26 | 320/256 | 1.5 | 4.0/0.4 |
|  |  | Oblique Coronal T2WI | 3378 | 102 | 30 | 320/256 | 1.5 | 4.0/1.0 |
|  |  | Oblique Axial DWI (b = 1000 s/mm2) | 2930 | 97 | 26 | 320/256 | 2 | 4.0/1.0 |
|  |  | Oblique Axial T1WI | 695 | Min | 26 | 320/256 | 1 | 4.0/0.4 |

**Supplementary Figures**


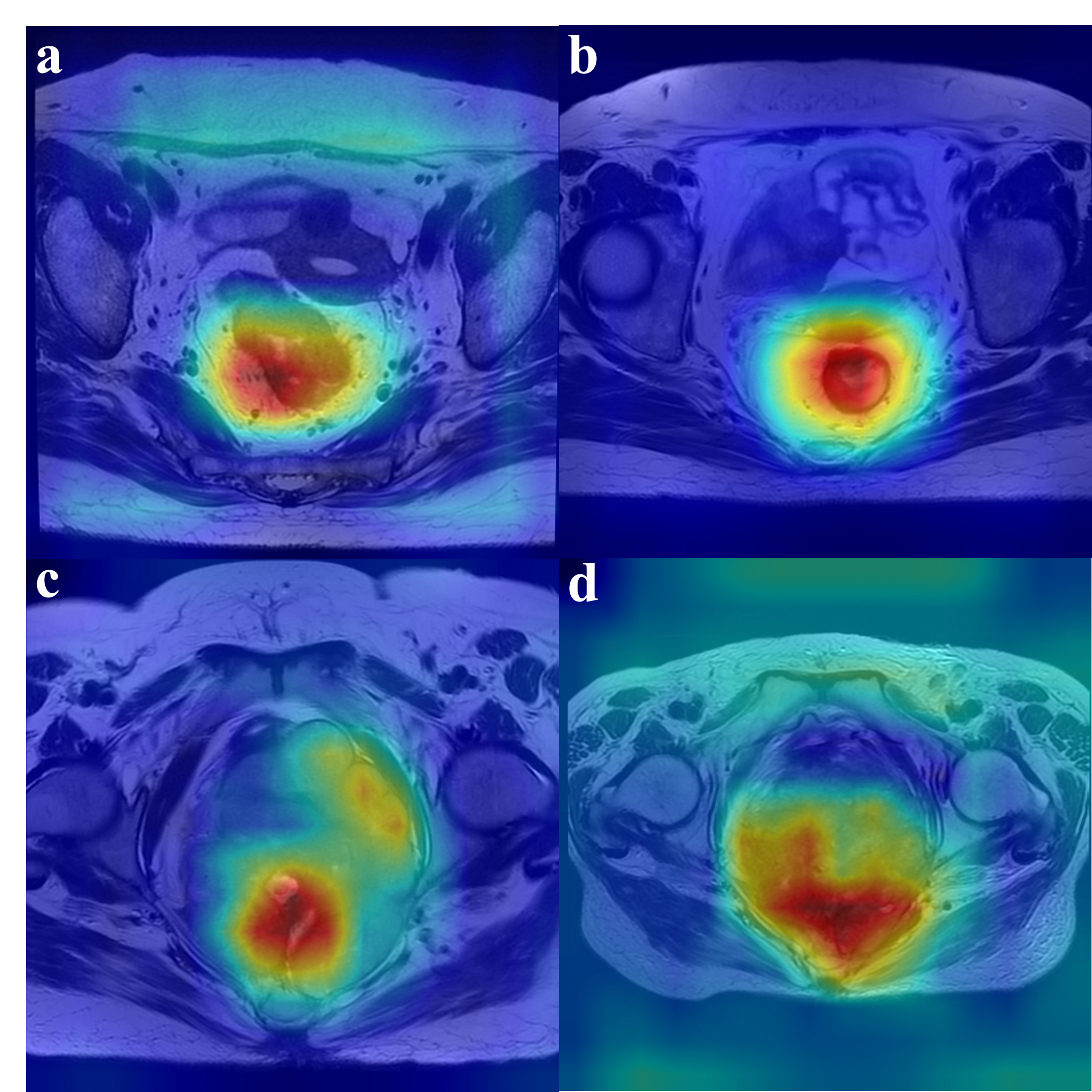


**Figure S1. Guided** **Grad-CAM results of the MR-DELTAnet for rectal cancer based on T2WI short-axis MRI before and after NCRT.** Red and yellow regions indicate areas of high attention by the MR-DELTAnet, with deeper red color representing higher contribution weights, whearas the blue regions indicate low or insignificant model attention. **(A)** Pre-treatment and **(B)** post-treatment Grad-CAM results of a 78-year-old women in the pCR group, showing the red regions persistently concentrated in the tumor core throughout treatment. **(C)** Pre-treatment and **(D)** post-treatment Grad-CAM results of a 70-year-old man in the non-pCR group; the pre-treatment red regions focus on the tumor, while post-treatment attention shifts to both the tumor and Peritumoral regions. Guided Grad-CAM = Guided Gradient-weighted Class Activation Mapping, pCR = pathological complete response NCRT = neoadjuvant chemoradiotherapy.


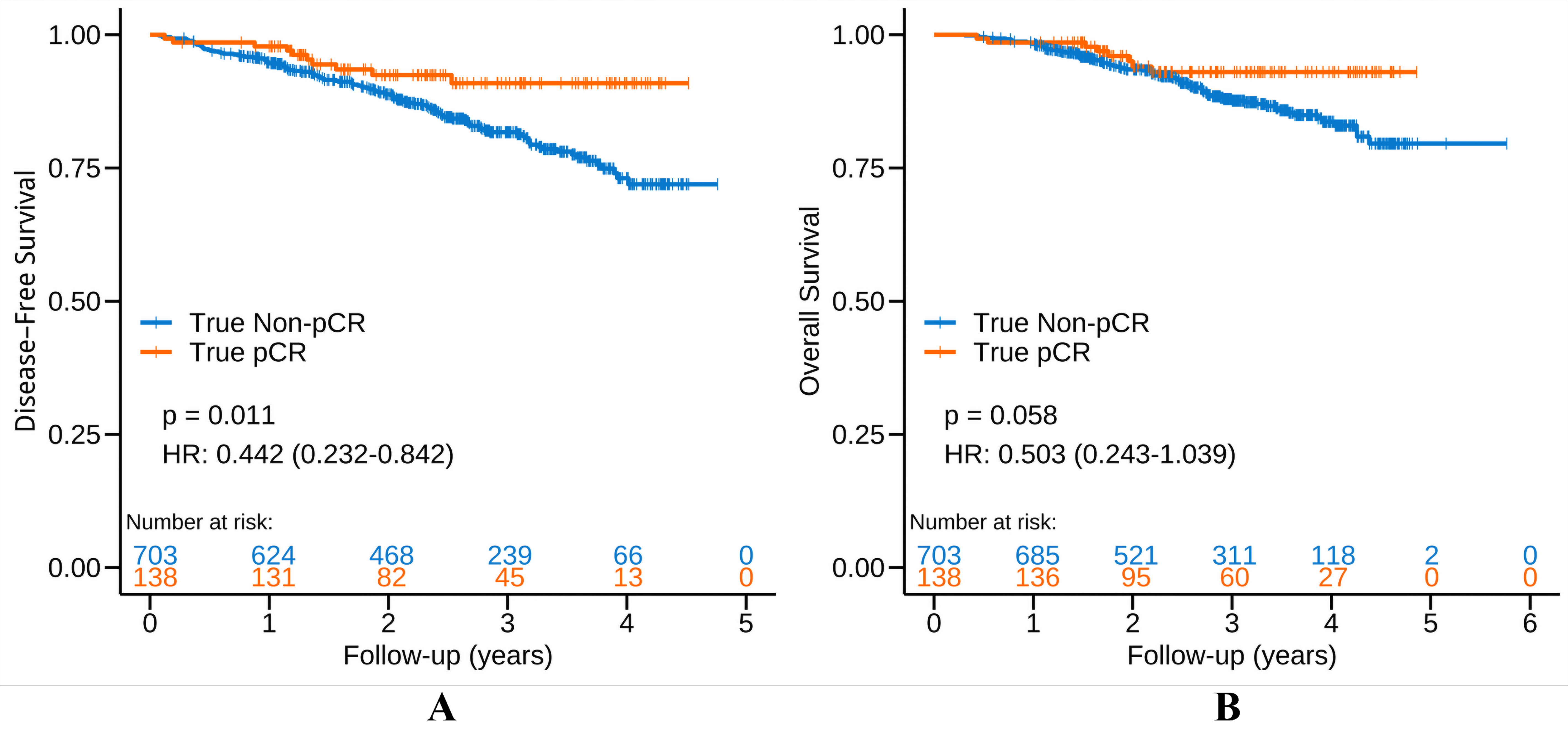


**Figure S2. Kaplan-Meier survival analysis.**Kaplan–Meier curves of diseases free survival (A) and overall survival (B) based on the pathology results


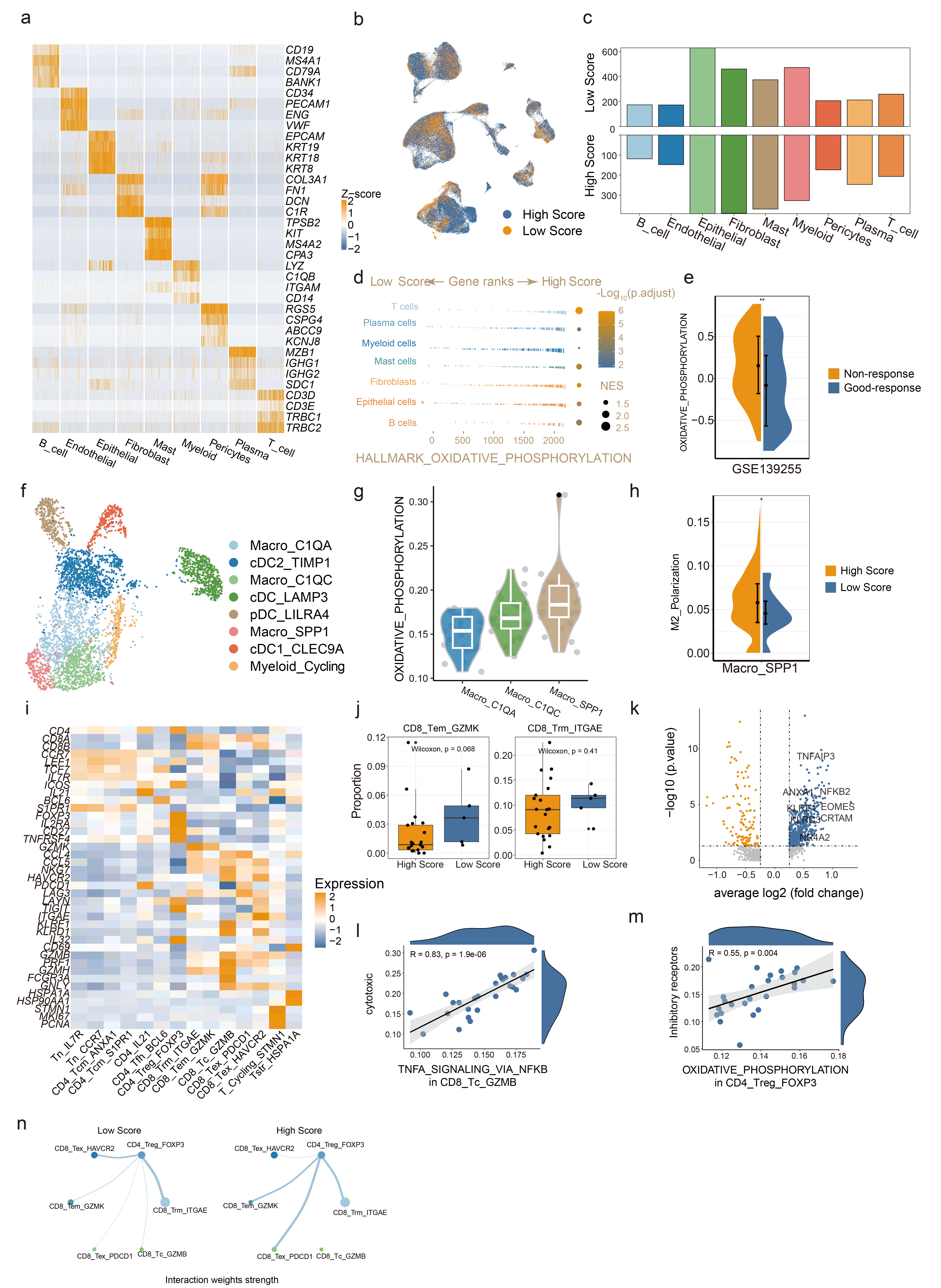


**Figure S3. The single cell level immune microenvironment is related to the MR-DELTAnet. (A)** The normalized expression of the marker genes of all cell types. **(B)** UMAP plot of groups of high- and low-score. **(C)** Barplot shows the number of different expressed genes of high- and low-score. **(D)** GSEA enrichment for oxidative phosphorylation in different cell types comparing high-score vs. low-score. Color of bubbles stands for -log10(p.adjust), size of bubbles stands for NES, normalize enrichment score. **(E)** Gene set scores of oxidative phosphorylation in GSE139255 dataset between response groups. **(F)** UMAP plot of the sub types of myeloid cells. **(G)** Gene set scores of oxidative phosphorylation among Macrophage subtypes. (**H)** Gene set scores of M2 Polarization in Macro_SPP1 between high- and low-score groups. **(I)** The normalized expression of the marker genes of the T cell sub types. **(J)** Proportion comparison of CD8_Tem_GZMK, and CD8_Trm_ITGAE between high- and low-score groups. Two-sided Wilcoxon test. **(K)** Volcano plot shows different expressed genes of high- and low-score in CD8_Tc_GZMB. Part of the genes with an average log2(fold change) > 0.5 and p.value < 0.05 are marked. **(L)** Dot plot shows the correlation between score of TNFa signaling via NFKB and cytotoxic score in CD8_Tc_GZMB among samples. Pearson correlation coefficient. **(M)** Dot plot shows the correlation between score of oxidative phosphorylation and Inhibitory receptors score in CD4_Treg_FOXP3 among samples. Pearson correlation coefficient. **(N)** Circle plot shows the interaction weights strength between CD4_Treg_FOXP3 and CD8+ T cells. NES = normalized enrichment score.
